# Child Myopia Prevalence in Europe: A Systematic Review and Meta-Analysis

**DOI:** 10.3390/children12060771

**Published:** 2025-06-13

**Authors:** Alicia Ruiz-Pomeda, Jose Luis Hernández-Verdejo, Pilar Cañadas, Noemi Guemes-Villahoz, Francisco Javier Povedano-Montero

**Affiliations:** 1 Optometry and Vision Department, Faculty of Optics and Optometry, Complutense University of Madrid, 28037 Madrid, Spain; jlhernan@ucm.es (J.L.H.-V.); pilarcanadas@ucm.es (P.C.); franpove@ucm.es (F.J.P.-M.); 2 Department of Ophthalmology, Instituto de Investigación Sanitaria del Hospital Clínico San Carlos (IdiSCC), C/Profesor Martin Lagos S/N, 28040 Madrid, Spain; noemi.guemes@salud.madrid.org; 3 Hospital Doce de Octubre Research Institute (i+12), 28041 Madrid, Spain

**Keywords:** myopia, prevalence, children, Europe

## Abstract

**Background/Objectives:** Information regarding the current myopia prevalence in children is limited. The aim of this study was to conduct a systematic review and meta-analysis to determine the prevalence of myopia in European children. **Methods:** A systematic review followed by a meta-analysis of relevant epidemiological studies published in the literature in children up to 18 years of age was performed. Web of Science, EMBASE and Scopus were searched from 2002 to 2022. **Results:** Of the 611 articles selected, 13 were included in the meta-analysis from 9 European countries with a sample size of 78,274 children, with a mean age of 8.2 years. The results suggested a trend of increasing myopia prevalence with age in most countries. France presented the highest myopia prevalence (19%) in children aged 9 years, while Denmark presented the lowest (0%) in children aged 4.5–7 years. Heterogeneity analysis indicated high heterogeneity (I^2^ 99.32%), suggesting significant variance in effect sizes among studies, with moderate dispersion (Tau 0.035) and a heterogeneity ratio H^2^ = 147.93. Egger’s test revealed funnel plot asymmetry (Z = 2.880, *p* = 0.004), while Kendall’s Tau (0.324, *p* = 0.076) was not statistically significant. The random-effect model estimated a combined weighted prevalence of myopia at 7.15% (95% CI: 4.3–10.0%), based on 78,274 participants. **Conclusions:** This meta-analysis provides comprehensive overview and current evidence on the prevalence of myopia in European children.

## 1. Introduction

Myopia is the most common refractive error worldwide [1] and its prevalence is increasing, particularly in East Asian countries [2], but also in Australia [3], the United States [4] and Europe among adults [5]. Myopia, especially high myopia, is associated with the development of ocular disorders such as myopic maculopathy, glaucoma, and retinal detachment among others. Children who develop myopia at an early age are at particular risk of these complications, which may lead to visual impairment and blindness, affecting educational performance and opportunities. This highlights the importance of managing myopia as a multifactorial condition that extends beyond mere visual impairment [6].

Previous meta-analyses have reported the prevalence of myopia in adults in different regions worldwide [1,5,7,8,9], but there are limited data on the prevalence of myopia in children.

An estimated 1.4 billion people were myopic in 2000, and the number is expected to reach 4.8 billion by 2050 [1]. It is well-known that the prevalence of myopia at different ages may vary significantly [10]. According to the World Health Organization (WHO) regions (Africa, Americas, Europe, Southeast Asia, and Western Pacific), the results of studies on the prevalence of refractive errors in children under 18 years of age between 1990 and 2016 showed that the estimated pooled myopia prevalence was 11.7% [95% confidence interval (CI): 10.5–13.0] [11].

Results from meta-analysis and systematic review revealed that the pooled prevalence of myopia in Chinese children is 38.0% [12]; among Indian children over the last four decades it was 7.5% (95% CI, 6.5–8.5%) in the 5–15-year age group and 10.7% (95% CI, 9–12.4) in the 11–15-year sub-group [13]; and among African children in the last two decades it was 4.7% (95% CI, 3.9–5.7) [9].

While systematic reviews and meta-analyses have been conducted on myopia prevalence in Asian, Indian, and African children, there is a noteworthy lack of robust data for Europe.

In 2022, the population in Europe reached a total of 556.6 million, with a total population of 9.68 million children aged 0 to 18 years [14].

There is a marked and widespread trend among children in developed countries to spend less time outdoors and more time performing near activities (reading, studying, or using computers and cell phones). Data suggest that not only genetic factors but also environmental factors [15,16,17] play an important role in the onset of myopia, and that these lifestyle and changes would probably explain the rapid increase in myopia in most of the world.

Epidemiological studies prior to 2011 have shown that, in white European populations, the prevalence of myopia was relatively low, affecting about 3–5% of 10-year-olds and up to 20% of 12–13-year-olds [18]. However, the prevalence of myopia in European children in the last decade remains unknown. Nevertheless, according to global projections, myopia rates are estimated to have increased significantly in Europe, potentially exceeding 40% [19,20]. This would imply that more than 3.5 million European children could already suffer from myopia, highlighting the growing concern about this vision disorder in Europe.

In this sense, the aim of our study is to assess the prevalence of myopia in European children by performing a comprehensive meta-analysis and systematic review from 2012 to 2022. The results may provide valuable information for appropriate preventive strategies to reduce the burden of myopia-related disorders in Europe.

## 2. Materials and Methods

This review strictly followed the guidelines established in the “Meta-analysis of Observational Studies in Epidemiology” for conducting systematic reviews and meta-analyses of observational studies [21] in addition to adhering to the Preferred Reporting Items for Systematic Reviews and Meta-Analyses (PRISMA) 2020 guideline and checklist [22]. This review was prospectively registered in the International Prospective Register of Systematic Reviews (PROSPERO) under the identifier ID 516894. The search was conducted in major databases, including Embase, Scopus, and the Web of Science Core Collection. For the search process, the following keywords, restricted to title, abstract, and keywords, were employed and combined using the Boolean operator OR: “prevalence,” “myopia,” “prevalence,” “ametropia,” “prevalence,” and “refractive error.”

To narrow the search to the specific population addressed in the study, the previous search was linked with the Boolean operator AND to a search that also utilized keywords restricted to the same fields: children or pediatric or adolescent or student. Finally, a country-specific filter was applied to each location on the European continent. The delimitation of European countries is primarily based on geographic location and the traditional definition of continental boundaries.

Only epidemiological studies reporting myopia prevalence among children up to 18 years old were included. Given the study’s aim to retrieve the most recent research, the temporal scope was limited to the years 2012–2022. For a better understanding, we describe the search process conducted:

(“prevalence” “myopia” (Topic) OR “prevalence” “ametropia” (Topic) OR “prevalence” “refractive error” (Topic)) AND (Children (Topic) OR Pediatric (Topic) OR Adolescent (Topic) OR student (Topic)), limited to 2002–2022.

### 2.1. Study Selection

At the initial stage, three reviewers (H-V. JL, R-P. A, and P-M. FJ) conducted independent assessments to evaluate the potential suitability of the studies based on their titles and/or abstracts. Subsequently, a comprehensive analysis of full articles was carried out, with a priority given to open-access articles and those strictly adhering to rigorous inclusion criteria. This meticulous search process was completed in October 2024.

A list of relevant studies was compiled, and after a detailed consensus process, studies that did not conform to the predefined criteria were excluded. Furthermore, procedures were employed to eliminate any duplicate records detected. Myopia was consistently defined across all studies as a spherical equivalent refraction of −0.50 D or less, as detailed in the table presented in the results section.

### 2.2. Data Extraction

The researchers independently conducted the extraction of necessary data from the selected articles, and these data were subsequently imported into a meticulously designed spreadsheet. This spreadsheet comprehensively recorded crucial details, such as the first author’s name, publication year, country of origin, average age of participants, total sample size, gender distribution, and myopia prevalence.

### 2.3. Statistical Analysis

All statistical analyses were conducted using Jamovi software (version 2.3.28). It should be noted that Jamovi is an open-source software developed in 2017 by Jonathon Love, Damian Dropmann, and Ravi Selker. Jamovi offers a range of advanced statistical analyses, including linear and logistic regression, analysis of variance, non-parametric tests, survival analysis, and meta-analysis. The choice of Jamovi was based on its well-established utility and accessibility in the scientific community.

## 3. Results

**Figure 1:**
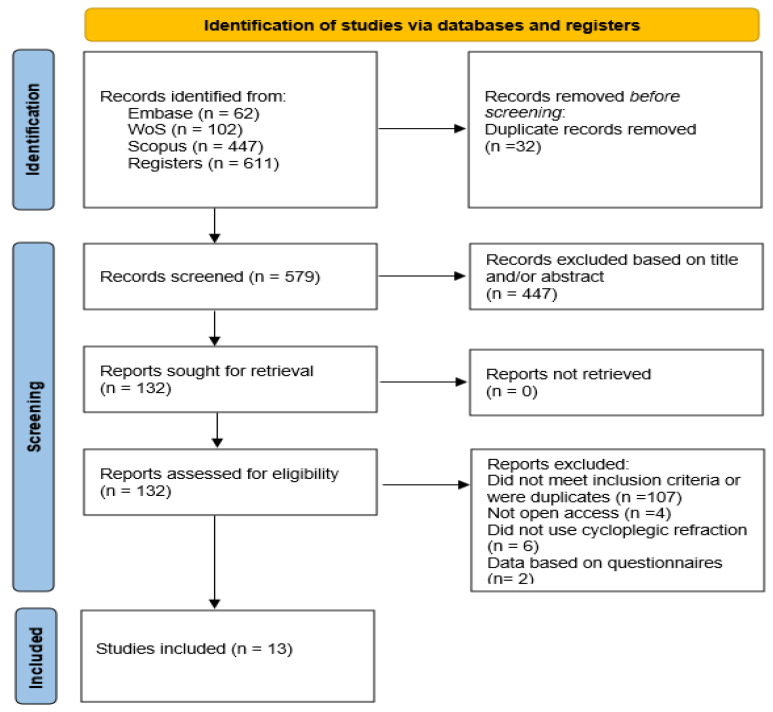
Selection search flow chart.

Figure 1 is a flowchart summarizing the study selection process, from the initial identification of records to the final inclusion. A total of 611 records were identified through comprehensive searches across multiple electronic databases. After the removal of 32 duplicate entries, 579 unique records underwent screening based on their titles and abstracts. Subsequently, 132 full-text articles were retrieved and assessed for eligibility. Following a detailed evaluation, 121 reports were excluded: 107 for not meeting the predefined inclusion criteria or being duplicate, 4 for not being open access [23,24,25,26], 6 for not using cycloplegic refraction [27,28,29,30,31,32] and 2 for questionnaire-based data [33,34]. The four studies excluded because they were not open access, presented methodological limitations or lacked access to full data. Finger, R.P. et al. [23] authored a letter to the editor rather than a research article and, therefore, did not provide original data on myopia prevalence. Nucci et al. [24] described objective refraction measurement in their methodology but did not report myopia prevalence, only data on visual impairment. Landmann A. et al. [25] was excluded because they estimated refractive error using a questionnaire rather than objective measurements, and the full text was not accessible through institutional credentials, preventing verification of the methodology and results. Finally, Popovic-Beganovic et al. (2018) [26] reported myopia prevalence based on cycloplegic autorefraction (20.4% among 997 children aged 7–16 years), but the full text was not available, preventing verification of methodology and results.

The final meta-analysis included 13 articles [35,36,37,38,39,40,41,42,43,44,45,46,47] from nine different countries. Detailed characteristics of the studies incorporated into the meta-analysis are depicted in Table 1. In seven of the articles, multiple datasets of myopia prevalence in different age groups were present. Therefore, these datasets were treated as separate studies for separate analyses. The overall sample size was 78,274 children, with a mean age of 8.2 years.

It is important to highlight that the studies included in this meta-analysis addressed the variable of age in heterogeneous ways. While some investigations focused on a narrow, specific age group, others reported data across broader age ranges. This methodological variability can influence the interpretation of prevalence rates across studies. Notably, some articles adopted a multi-age-group approach, among which the study by Demir et al. [44] stands out for its exceptionally broad age coverage, ranging from 8 to 16 years. To ensure consistency and comparability in the statistical analysis, mean age values were calculated for studies that reported data across multiple age brackets.

**Figure 2:**
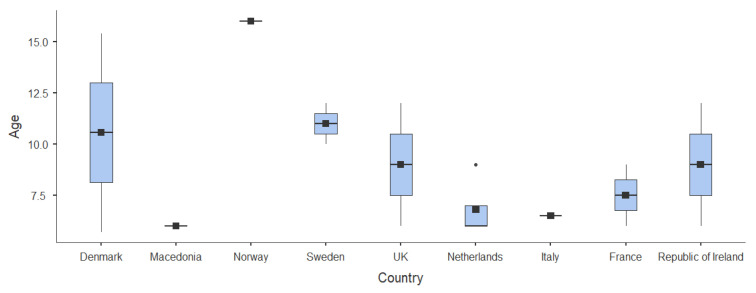
Age ranges analyzed in the different studies.

Figure 2 provides a visual representation of the age distributions across countries, clearly illustrating the variability in the age ranges assessed among the included studies.

A random-effect meta-analysis was conducted using the Restricted Maximum-Likelihood (REML) estimator to compute the combined prevalence of myopia from 13 studies conducted in nine European countries, all of which used cycloplegic refraction. The combined prevalence estimate from the model was 7.15% (95% Confidence Interval: 4.3% to 10.0%), with a statistically significant model intercept (Z = 4.90, *p* < 0.001). The total sample included 78,274 children, with considerable variability in the mean age across studies, ranging from 6.0 to 16.0 years, as shown in Figure 2.

**Figure 3:**
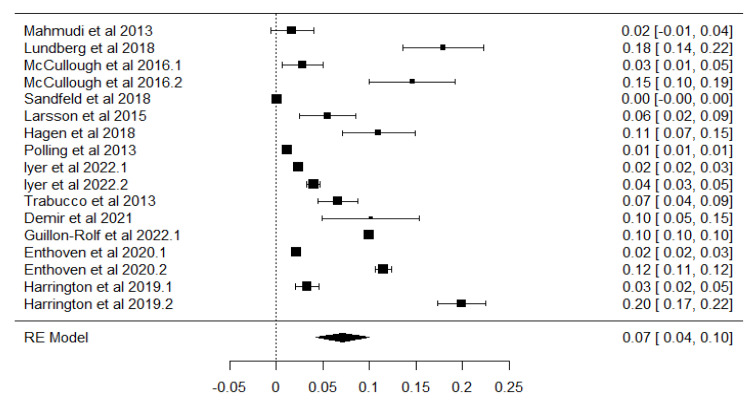
Forest plot chart for all selected studies included in the meta-analysis [35,36,37,38,39,40,41,42,43,44,45,46,47]. The black squares represent individual study effect sizes and 95% confidence intervals, and the dotted vertical line indicates the pooled estimate.

The analysis revealed substantial between-study heterogeneity, with a tau-squared (τ^2^) value of 0.0034 (Standard Error = 0.0013), corresponding to a tau (√τ^2^) of 0.059. The I^2^ statistic indicated that 99.74% of the total variability was due to heterogeneity rather than sampling error. This was further supported by Cochran’s Q test (Q = 3612.49, df = 16, *p* < 0.001), and the heterogeneity ratio (H^2^) was 387.32. These results highlight significant methodological and demographic differences among the included studies, justifying the use of a random-effect model. The individual study estimates, along with their confidence intervals and the pooled effect size, are graphically presented in Figure 3, which displays the forest plot summarizing the prevalence of myopia across the included studies.

**Figure 4:**
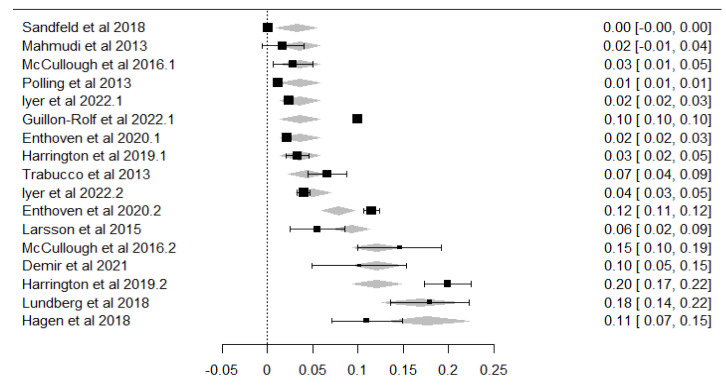
Bubble plot of meta-regression showing the association between age and myopia prevalence. Each square represents a study estimate with 95% confidence interval; the dotted line and grey area indicate the predicted effect and its 95% confidence region. References shown: [35,36,37,38,39,40,41,42,43,44,45,46,47].

To explore potential sources of heterogeneity, a meta-regression analysis was performed using the mean age of participants as a continuous moderator (Figure 4). The model revealed a statistically significant association between age and myopia prevalence, with a positive regression coefficient of 0.0141 (Standard Error = 0.00279; Z = 5.04, *p* < 0.001; 95% CI: 0.009 to 0.020). This suggests that, on average, each additional year of age was associated with a 1.41% increase in the estimated prevalence of myopia across studies.

Although residual heterogeneity remained high, the model accounted for a substantial portion of the between-study variance. The heterogeneity statistics were τ^2^ = 0.0013 (SE = 0.0005), τ = 0.035, and I^2^ = 99.32%, with a heterogeneity ratio H^2^ = 147.93. Importantly, the meta-regression model explained 63.46% of the total heterogeneity (R^2^), confirming that mean age is a meaningful explanatory variable for the observed variation in prevalence estimates.

**Figure 5:**
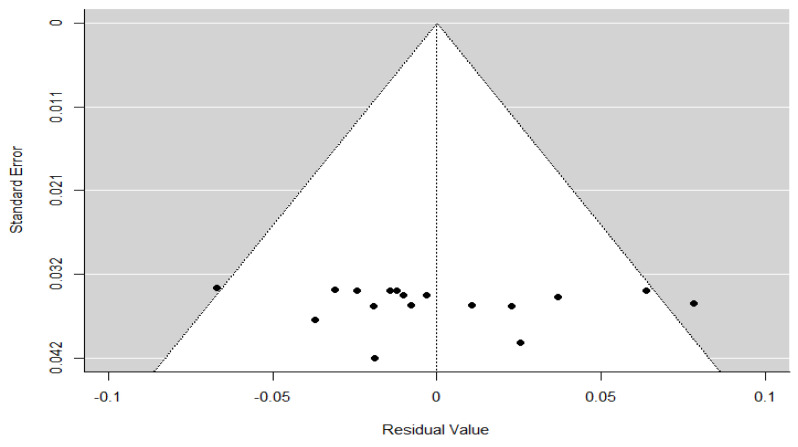
Funnel plot of the meta-regression model. Each black dot represents an individual study. The grey shaded area represents the 95% confidence region, and the vertical dotted line indicates the pooled effect estimate under the meta-regression model.

To assess the potential presence of publication bias, a funnel plot was obtained/performed (Figure 5) using the residual values from the meta-regression model with age as a continuous moderator. Visual inspection of the funnel plot showed a symmetric distribution of studies around the mean effect size, with no clear evidence of asymmetry. The data points were evenly spread within the pseudo 95% confidence limits, with no indication of publication bias or small-study effects.

This visual impression was confirmed by formal statistical tests: Kendall’s rank correlation test yielded Tau = 0.324 (*p* = 0.076), which was not statistically significant, and Egger’s regression test reported Z = –0.487 (*p* = 0.627), indicating no significant asymmetry. Additionally, the Fail-safe N analysis estimated that 14,657 null-result studies would be required to nullify the observed effect (*p* < 0.001). Taken together, these findings suggest a low risk of publication bias, although caution is warranted due to the high heterogeneity among the included studies.

**Figure 6:**
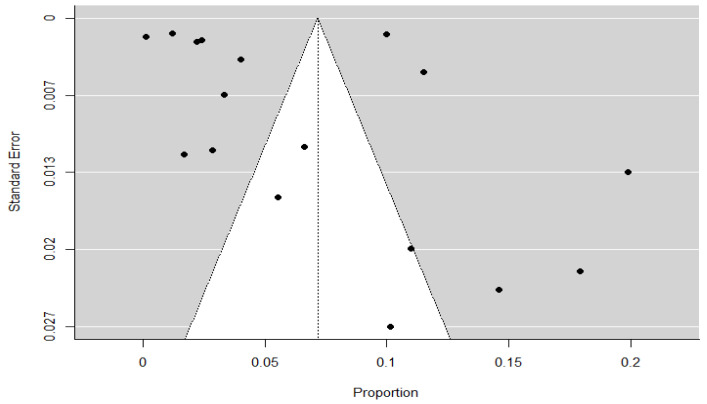
Funnel plot for the overall meta-analysis. Each black dot represents an individual study included in the analysis. The vertical dotted line indicates the pooled effect size, and the grey triangular region represents the expected 95% confidence region in the absence of publication bias.

To evaluate the presence of publication bias in the overall meta-analysis, a funnel plot was generated for all included studies (Figure 6). Visual inspection of the plot revealed slight asymmetry, with a mild concentration of studies on the left side of the mean effect. This visual impression was confirmed by Egger’s regression test for funnel plot asymmetry (Z = 2.880, *p* = 0.004), indicating statistically significant asymmetry. In contrast, the rank correlation test using Kendall’s tau did not reach statistical significance (τ = 0.324, *p* = 0.076), suggesting a less conclusive result.

Additionally, the Fail-safe N analysis using Rosenthal’s method yielded a value of 14,657 (*p* < 0.001), indicating that many additional null-result studies would be required to nullify the observed effect. Taken together, these findings suggest a low to moderate risk of publication bias, which should be considered when interpreting the pooled prevalence estimates.

## 4. Discussion

Myopia is recognized as one of the leading global public health challenges, with increasing prevalence observed in countries such as Australia, the United States, and East Asian countries [8]. This meta-analysis included 13 epidemiologic studies conducted in European countries, comprising a total sample of 78,274 children with a mean age of 8.2 years. The temporal scope of the review was narrowed to studies published between 2002 and 2022. Only studies that reporting on the prevalence of myopia in European children up to 18 years of age and using cycloplegic refraction were included. The exclusion of non-cycloplegic studies was necessary, as non-cycloplegic refraction in children tends to overestimate the degree of myopia due to active accommodation [48,49].

Given that the mean age of participants varied across the included studies, additional analyses were performed to account for differences in age distribution.

This meta-analysis showed that there is a trend of increasing myopia prevalence with age in most countries. According to Table 1, the France and Denmark had the highest and lowest prevalence of myopia, respectively (19% vs. 0%). The 0% prevalence reported in Denmark comes from a study of 445 Danish children aged 4.5–7 years [38], whereas in the same country, another study with a sample of 307 subjects between 14 and 17 years showed a prevalence of 17.9% [36]. The highest prevalence, 19%, was observed in a French population of 48,163 subjects aged 9 years [29]. Similar results have been found in other meta-analyses of prevalence in Chinese children, showing that the prevalence of myopia is strongly associated with older age [8]. This pattern, consistent in many countries, suggests that myopia develops as children grow older, and is influenced by both genetic and environmental factors. It is well known that from birth, refractive error gradually shifts towards emmetropia as the eye grows. However, in some individuals, this process can deviate towards myopia, which may progress with age due to factors that remain unclear [15,16,17]. The Danish study reported a 0% prevalence in children aged 4.5–7 years. However, this estimate should be interpreted with caution due to the exclusion of children from special kindergartens and a participation rate below 50%, which may have led to selection bias.

The prevalence of myopia in children aged 6–7 years shows relatively consistent values across several European countries, except for France. For instance, prevalence rates were 1.9% in Northern Ireland [37], 3.3% in the Republic of Ireland [47], and 2.2%, 2.4%, and 4% in three studies conducted in The Netherlands [41,42,46]. In contrast, a prevalence of 10% was reported in France [45]. As all these studies used cycloplegic refraction, methodological differences are unlikely to account for the disparity. The higher prevalence observed in the French study may instead be related to other factors, such as sample characteristics, urbanization level, or lifestyle differences affecting near work and outdoor time.

Our results show higher myopia rates in older age groups, for example, in Sweden, the prevalence among children aged 8–16 years was 10% [44]; in Norway, it was 11% at age 16 [40]; and in Denmark, it was 17.9% among those aged 14–17 years [36].

The positive association between age and myopia prevalence observed in our meta-regression analysis is consistent with prevalence data reported in individual European studies. These findings support the trend identified in the meta-regression, where each additional year of age was associated with a 1.41% increase in myopia prevalence. This age-related increase likely reflects the progressive onset and development of myopia during childhood and adolescence, a pattern that has important implications for screening strategies and early interventions. Although global projections estimate that the number of people with myopia will triple from 1.4 billion in 2000 to 4.8 billion by 2050 [1], current European data for children under 10 years show considerably lower rates. Obtaining cycloplegic, population-based data for adolescents and young adults in Europe is crucial to monitor this projected increase.

Other aspects to consider in the diverse results obtained in our study between countries are ethnicity, geographical area (urban vs. rural) or lifestyle factors. Indeed, previous research has shown that the prevalence of myopia varies significantly across ethnic groups [50]. While we present prevalent data from different European countries, not all the studies provide detailed information on these aspects. In Denmark, Sandfeld et al. [38] focused on urban populations and Lundberg et al. [36] examined physical activity. In Sweden, Demir et al. [44] explored both genetic and environmental factors affecting myopia development, although it was not specified whether there were regional differences, while Larsson’s et al. [39] researched concentrated on urban populations. In The Netherlands, Iyer et al. [42] and Polling et al. [41] did not specify the type of population studied, whereas Enthoven et al. [46] highlighted the implications of urban environments, particularly concerning prolonged screen time.

Variations may reflect genetic, environmental and lifestyle influences, such as time spent outdoors, which has been shown to have a protective effect against the development of myopia [51]. The main clinical indicator of genetic predisposition, without genetic testing, is the family history of myopia [52]. Recent research, beyond those earlier studies, revealed a substantial link between the onset of myopia and the number of parents with myopia. This connection has been succinctly outlined in a recent meta-analysis [53]. Additional factors should be considered regarding the role of parental myopia as a risk factor. Two primary environmental factors influencing the onset of myopia include amount of time spent outdoors and near-work activities. The potential protective effect of outdoor activities against the development of myopia is still not fully understood. Although there are some hints that near work may not have a direct correlation with myopia, more recent findings indicate a discernible connection [54]. The term “near work” includes a broad range of factors like education level, indoor study hours, reading distance, font size, and screen-related activities; therefore, the quantification of its actual impact is complex. Nevertheless, different studies and meta-analyses revealed that increased near-work time is linked to a higher risk of myopia and supports the connection between higher extent of near work and the onset and progression of myopia [51,54,55,56,57]. Evidence from longitudinal studies, especially from East Asia, suggests that schooling plays a critical role in refractive development, with myopia onset and progression strongly associated with academic exposure. This factor should be considered in future European studies examining myopia progression. French et al. [58] found that 6-year-old children with limited outdoor exposure and substantial near work were at risk of developing myopia at age 12 years of age, in contrast to those with those who spend more time outdoors and have limited near-work time. Moreover, urban or rural living have also been related with myopia progression [54]. These findings underscore the importance of incorporating strategies that promote outdoor activities and reduce near work, especially in urban settings, to mitigate the growing prevalence of myopia among children.

The prevalence of myopia by sex showed different trends across countries. In Denmark, a higher prevalence was found in boys 20.5% vs. 15.1% in girls at age 16 [36]. In Northern Ireland, prevalence increased with age in both sexes, with a slightly higher prevalence in girls at age 12 [37]. In Norway the prevalence of myopia in girls at 16 years of age was more than twice as high as in boys (14.4% vs. 6.5%) [40]. Finally, in Italy, the prevalence at 6.5 years was higher in boys (4%) than in girls (2.6%) [43]. These differences may be related to possible socio-environmental influences, such as outdoor time or near work, suggesting that there is no connection between sex and myopia, and the prevalence can vary over time. However, some studies performed in China and Saudi Arabia [59,60,61], showed higher myopia prevalence in females than in males.

The assessment of publication bias yielded mixed findings. While the funnel plot based on the meta-regression residuals appeared symmetrical and formal tests did not detect significant asymmetry, the funnel plot from the overall meta-analysis showed slight asymmetry, supported by a statistically significant result in Egger’s test. This suggests that some degree of publication bias may be present, although the large Fail-safe N value indicates that the observed effect is robust and unlikely to be overturned by unpublished studies with null results. These trends highlight the need to promote the publication of negative or neutral results and to implement editorial policies that support a more balanced and comprehensive representation of available evidence. Therefore, although the overall risk of bias appears low to moderate, these findings warrant caution when interpreting the pooled prevalence estimates.

## 5. Conclusions

This is the first systematic review and meta-analysis of the prevalence of myopia in European children. In conclusion, our meta-analysis presented the prevalence results from 13 epidemiological studies of nine different countries. The combined prevalence estimate of myopia in European children was 7.15% (95% CI: 4.3% to 10.0%), based on data from 78,274 participants across the studies. Despite the substantial heterogeneity observed, the meta-regression analysis identified mean age as a significant moderator, explaining 63.46% of the total between-study variability. These findings highlight age as a key factor influencing myopia prevalence and underscore the importance of considering age distribution when interpreting epidemiological data and designing vision screening programs.

Our study has some limitations. Firstly, while the definition of myopia was rather consistent across the studies analyzed, none of them specified the maximum range of myopia or made a distinction between myopia and high myopia. These inter-study variations in the definitions of myopia and high myopia may have influenced the results.

Another important aspect not addressed by the studies included was the geographical and ethnic variations. It is recognized that myopia prevalence may differ by geographical location and ethnic backgrounds of children [2,6,50,57], which, if not adequately considered, might introduce biases into the meta-analysis. In this sense, one of the main limitations of this meta-analysis is the unequal geographic distribution of the included studies. While our systematic search aimed to cover all European countries without restriction, high-quality, population-based studies using cycloplegic refraction were only available from a limited number of countries, particularly in Northern and Western Europe. This imbalance reflects the current state of the literature rather than a selection bias in our methodology and highlights the need for further epidemiological research in Southern and Eastern European countries using standardized and reliable protocols. Environmental factors such as sunlight exposure or climate were not analyzed in this study, although they may be considered in future research on myopia risk factors.

The variability in the age ranges across studies poses challenges in making direct comparisons between countries. While age-specific prevalence data are essential for tracking myopia trends, many European studies report prevalence across broad age groups. To improve comparability, future research should aim to provide more age-specific prevalence data.

We have also identified a certain publication bias, where studies with positive or significant results might be more likely to be published, potentially skewing the overall estimation of myopia prevalence. Considering these limitations is crucial for interpreting the results of a myopia prevalence meta-analysis in European children and understanding the reliability and validity of the estimates obtained.

In summary, this meta-analysis provides thorough and up-to-date evidence regarding myopia prevalence among European children. For more accurate comparisons and analysis, it would be beneficial to have consistent age ranges across countries. This would be useful to better understand the context of these prevalence rates. Monitoring myopia trends over time and identifying influential factors, especially in populations experiencing shifts in myopia, is crucial. Further understanding the causes of childhood myopia can guide prevention efforts, potentially providing strategies to reduce the economic burden associated with refractive errors. However, the findings should be interpreted with caution, as they reflect the prevalence of myopia only in the European countries for which high-quality, population-based cycloplegic data are currently available.

## Figures and Tables

**Table 1:** Characteristics of the studies included in the review.

Author/Year	Country	Sample	Age	P Total	P Boys	P Girls	Cycloplegia Refraction	Myopia
		N	Years	%	%	%	Drops	EE (D)
Mahmudi et al., 2013 [35]	North Macedonia	119	3–9	1.6	NA	NA	(ND)	−0.50
Lundberg et al., 2018 [36]	Denmark	307	14–17	17.9	20.5	15.1	Tropicamide	−0.50
Sandfeld et al., 2018 [38]	Denmark	445	4.5–7	0	ND	ND	Cyclopentolate	−0.50
Harrington et al., 2019 [47]	Ireland	728	6–7	3.3	NA	NA	(ND)	−0.50
McCullough et al., 2016 [37]	UK (Northern Ireland)	212 (4)	6–7	1.9	1	2.8	Cyclopentolate	−0.50
		226 (37)	12–13	16.4	15.5	17.1		
Demir et al., 2021 [44]	Sweden	128	8–16	10	ND	ND	Cyclopentolate	−0.50
Larsson et al., 2015 [39]	Sweden	217	10	5.5	ND	ND	Cyclopentolate	−0.50
Hagen et al., 2018 [40]	Norway	246	16	11	6.5	14.4	Cyclopentolate	−0.50
Iyer et al., 2022 [42]	The Netherlands	6934	6	2.4	ND	ND	ND	−0.50
		2974	7	4	ND	ND		
Enthoven et al., 2020 [46]	The Netherlands	5021	6	2.2	ND	ND	Cyclopentolate	−0.50
		4709	9	11.5	ND	ND		
Polling et al., 2013 [41]	The Netherlands	6690	6	1.2	ND	ND	ND	−0.50
Trabucco et al., 2013 [43]	Italy	500 (33)	3–10	6.6	4	2.6	ND	ND
Guillon-Rolf et al., 2022 [45]	France	48,163	6	10	ND	ND	Cyclopentolate	−0.50
			9	19	ND	ND	Cyclopentolate/Atropine	

ND not described.

## Data Availability

The data presented in this study are available on request from the corresponding author.
